# Optical coherence tomography findings as a predictor of clinical course in patients with branch retinal vein occlusion treated with ranibizumab

**DOI:** 10.1371/journal.pone.0199552

**Published:** 2018-06-20

**Authors:** Akira Shiono, Jiro Kogo, Hiroki Sasaki, Ryo Yomoda, Tatsuya Jujo, Naoto Tokuda, Yasushi Kitaoka, Hitoshi Takagi

**Affiliations:** Department of Ophthalmology, St. Marianna University School of Medicine, Kawasaki, Kanagawa, Japan; National Yang-Ming University Hospital, TAIWAN

## Abstract

**Purpose:**

To examine the relationship between optical coherence tomography (OCT) images and clinical course in eyes with branch retinal vein occlusion (BRVO) treated with intravitreal ranibizumab injection (IVR).

**Design:**

Prospective cohort study.

**Participants:**

Thirty eyes of 30 patients with BRVO treated with IVR.

**Methods:**

All patients received 1 initial IVR followed by repeated injections in the pro re nata (PRN) regimen. Correlations between logarithm of minimum angle of resolution best-corrected visual acuity (logMAR BCVA) or number of IVRs after 12 months and OCT parameters including the external limiting membrane (ELM), ellipsoid zone (EZ), interdigitation zone (IZ), and photoreceptor outer segment (PROS) length at first resolution of macular edema (ME) were assessed. Resolution of ME was defined as central foveal thickness <300 μm and the absence of subretinal fluid. OCT parameters influencing BCVA and number of IVRs were evaluated using multivariate analysis. Correlations between nonperfusion areas (NPAs) and thinning areas and changes in retinal thickness of BRVO-affected areas were assessed.

**Results:**

Of the 30 patients, 27 completed this study and were included in the statistical analyses. The mean logMAR BCVA at 3, 6, and 12 months was 0.16 ± 0.19, 0.09 ± 0.20, and 0.07 ± 0.20, respectively, which improved significantly from baseline at each visit (p < 0.0001, respectively), while the mean number of IVRs at 12 months was 3.9 ± 2.2. The mean number of IVRs for the first resolution of ME was 1.6 ± 0.8. Eyes with ELM and EZ defects at the points of first resolution of ME were correlated with a significantly lower BCVA at 12 months compared with eyes with preserved ELMs and EZs (p = 0.035, p = 0.002, respectively). However, eyes with IZ defects at the points of first resolution of ME were not correlated with a significantly lower BCVA at 12 months compared with eyes with preserved IZs (p = 0.160). Defects in the EZ at the points of first resolution of ME significantly affected the number of IVRs at 12 months (p = 0.042), although the ELM and IZ did not. PROS length at the points of first resolution of ME was significantly correlated with BCVA and number of IVRs at 12 months (p = 0.006, p = 0.0008, respectively). In multivariate analysis, PROS length at the points of first resolution of ME had the most significant effect on BCVA and number of IVRs (p = 0.013, p = 0.012, respectively). NPA size on fluorescein angiography and thinning area on OCT within the macular area showed a significant correlation (p = 0.003, r = 0.971). The retinal thickness of ischemic BRVO-affected areas was significantly less than that of control areas at 10, 11, and 12 months (p = 0.001, p = 0.005, p = 0.003, respectively).

**Conclusion:**

We showed that the 1+PRN regimen may be a useful therapy for ME due to BRVO. In addition, PROS length at points of first resolution of ME appears to be a good indicator of BCVA and number of IVRs in BRVO patients.

## Introduction

Branch retinal vein occlusion (BRVO) is a retinal vascular disease and cause of visual loss due to macular edema (ME) and retinal ischemia. The Branch Vein Occlusion Study (BVOS) reported that grid laser photocoagulation increased visual acuity in patients with ME due to BRVO[1]. However, that effect was limited. In 2009, the Standard Care vs Corticosteroid for Retinal Vein Occlusion (SCORE) study showed that intravitreal triamcinolone administration resulted in similar visual improvement but with high rates of intraocular pressure elevation[2]. Recently, anti-vascular endothelial growth factor (VEGF) has been used for the treatment of ME due to RVO, with greater increases in visual acuity compared with laser photocoagulation[3–8].

Several large clinical trials were conducted in which anti-VEGF injections were administered monthly for at least 6 months. After the initial treatment period, anti-VEGF injections were administered on an as-needed basis. Anti-VEGF treatment resulted in marked improvement in visual acuity in patients with BRVO. However, some patients with BRVO-reduced visual acuity continued to require intravitreal anti-VEGF injections, and anti-VEGF therapy is associated with several problems such as the risk of endophthalmitis, retinal detachment, and cardiovascular events as well as cost. Recently, Miwa et al. have reported that one initial intravitreal ranibizumab injection (IVR) and 3 monthly IVR regimens achieved similar functional outcomes at 12 months[9]. Consequently, loading-dose ranibizumab injections in the treatment of ME due to BRVO may be unnecessary.

Previously, several groups including ours[10–17] reported that spectral domain optical-coherence tomography (SD-OCT) findings were indicators of visual function and prognostic factors in patients with macular disease. OCT is a well-established diagnostic and monitoring tool for vitreomacular disorders. Thus, the clinical course of patients with BRVO treated with IVR may be predicted by OCT findings.

The aim of this study was to examine the relationship between OCT images and clinical findings in eyes with BRVO treated with 1 initial IVR followed by a monthly pro re nata (PRN) regimen (1+PRN).

## Methods

This prospective study was approved by the Institutional Review Board of St. Marianna University of Medicine (Kanagawa, Japan), and adhered to the tenets of the Declaration of Helsinki. Study participants provided written informed consent. We registered this study in Clinical Trial Government (https://clinicaltrials.gov) (ClinicalTrials.gov Identifier: NCT02144662, registered on March 22, 2014). Thirty patients with ME due to BRVO were enrolled in this study. One death occurred in the 1-year study period, and 2 patients dropped out. Statistical analyses were performed on the results of the 27 patients who completed this study. The BRVO diagnosis was based on fundus examination and fluorescein angiography (FA) findings. Key inclusion criteria were: treatment-naïve patients; age >18 years; ME due to BRVO involving the fovea; and macular thickness >300 μm at the initial visit. Key exclusion criteria were: central retinal vein occlusion; hemi-central retinal vein occlusion; and other chorioretinal disease such as diabetic retinopathy, hypertensive retinopathy, and choroidal neovascularization.

### Treatment

All patients received 1 initial IVR followed by a monthly PRN regimen without a loading phase. At monthly visits, repeat injections were administered if there was evidence of ME (mean central foveal thickness [CFT] >300 μm) or subretinal fluid (SRF) at the fovea on OCT images. Eyes with nonperfusion areas (NPAs) >10 optic disc areas (DAs) in size on FA were determined to represent ischemic BRVO, and scatter laser photocoagulation was performed on those eyes at 12 months.

### Examination

**Fig 1:**
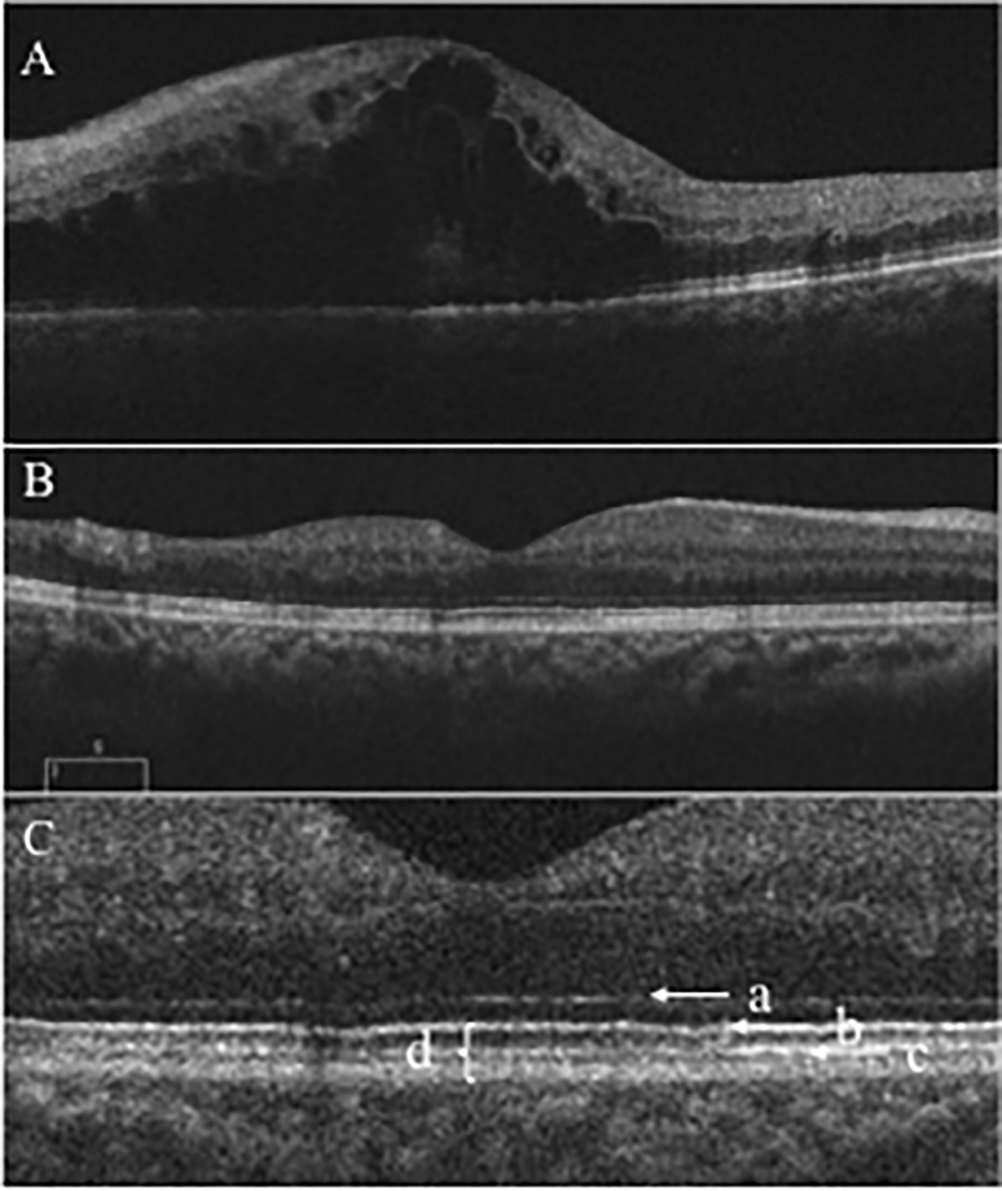
Optical coherence tomography (OCT) image before and after intravitreal ranibizumab injection (IVR). (A) OCT image before IVR. The outer retina was not detected. (B) OCT image at the point of resolution of ME. (C) Enlargement of the OCT image showing the ELM (a), EZ (b), IZ (c), and PROS length (d). ELM = external limiting membrane; EZ = ellipsoid zone; IZ = interdigitation zone; PROS = photoreceptor outer segment.

According to the study protocol, patients were scheduled for monthly monitoring visits during which they underwent a complete visual examination including measurement of the mean logarithm of minimum angle of visual acuity (logMAR) BCVA with a Landolt chart, determination of intraocular pressure, and OCT (Cirrus HD-OCT, Carl Zeiss Meditec, Dublin, CA). Macular morphologic evaluation was determined based on OCT images at each visit. CFT was defined as the mean distance between the inner limiting membrane (ILM) and retinal pigment epithelium (RPE) within a central subfield. CFT measurements were derived from the software (Cirrus 3.0; Carl Zeiss Meditec, Inc.) provided by the manufacturer. Examination of the outer retina including external limiting membrane (ELM), ellipsoid zone (EZ), interdigitation zone (IZ), and photoreceptor outer segment (PROS) length were examined with the Cirrus HD-OCT using 5-line raster scans. PROS length measurement was described in our previous report[15] (Fig 1). To assess retinal perfusion status, all patients underwent FA (Spectralis HRA+OCT, Heidelberg Engineering, Heidelberg, Germany) at 12 months.

The study aimed to identify prognostic factors for the improvement of BCVA and number of IVRs at 12 months, such as OCT parameters of the outer retina at the points of the first resolution of ME. These were examined using multivariate analysis. Resolution of ME was defined as CFT <300 μm and the absence of SRF.

**Fig 2:**
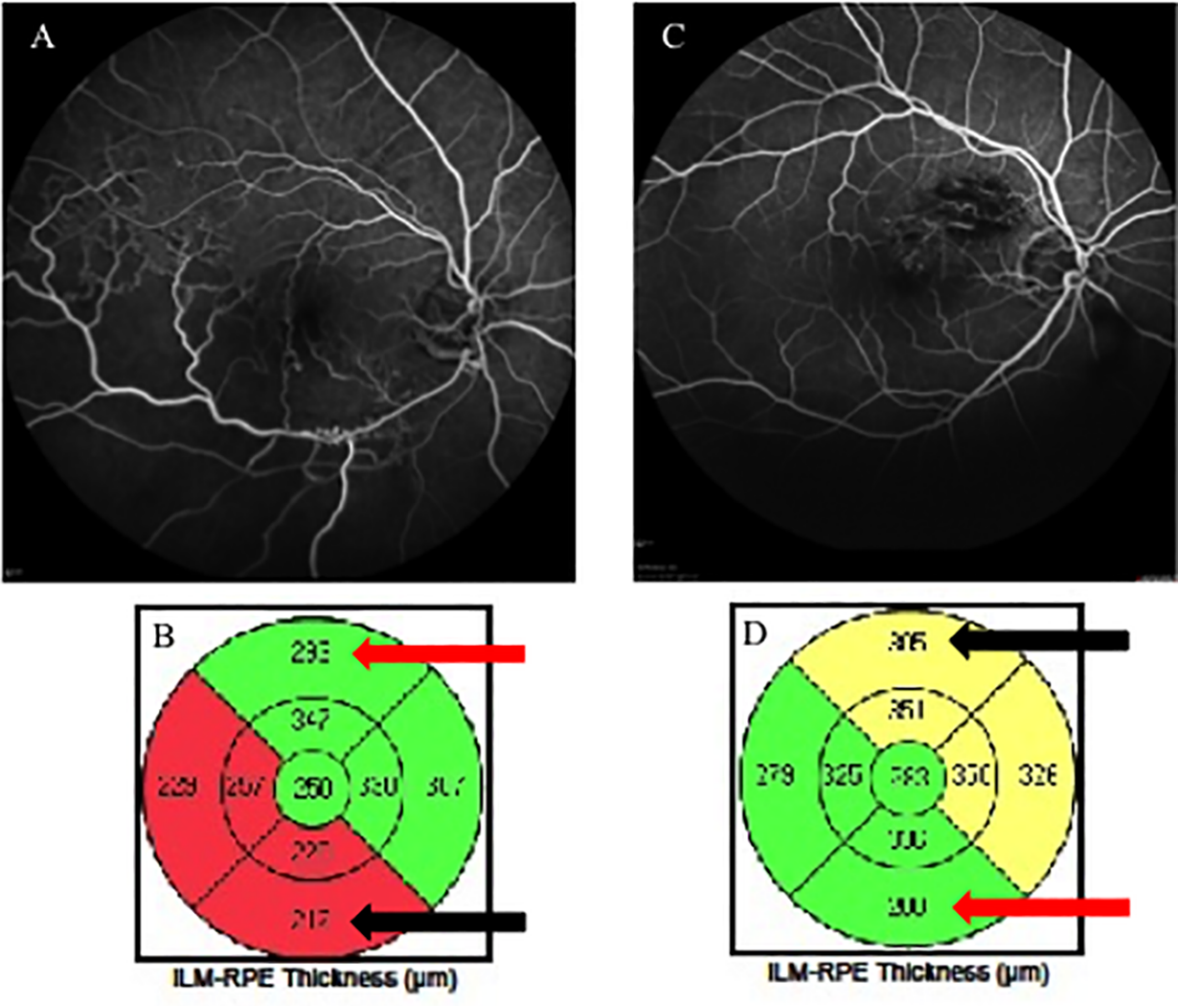
Fluorescein angiography (FA) images and macular cube analysis of branch retinal vein occlusion (BRVO). (A, B) FA images and macular cube analysis of ischemic inferotemporal BRVO. These parameters show the retinal thickness of each sector within the macular area as calculated automatically. The retinal thickness of the BRVO-affected area is shown in the lower section (black arrow), and a symmetrical area was examined to determine the retinal thickness of the control area (red arrow). (C, D) FA images and macular cube analysis of nonischemic superotemporal BRVO. In this case, the retinal thickness of the BRVO-affected area is shown in the upper section (black arrow), and a symmetrical area was examined to determine the retinal thickness of the control area (red arrow).

**Fig 3:**
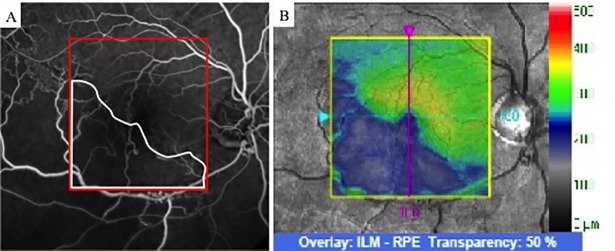
Fluorescein angiography (FA) of ischemic branch retinal vein occlusion (BRVO). (A) FA image of ischemic BRVO. The nonperfusion area is outlined in white. The area indicated by the red square was identical to the OCT image obtained by macular cube scan (200 × 200) (right, indicated by the yellow square). (B) OCT image showing a layer map of retinal thickness. The thinning area is indicated in blue. OCT = optical coherence tomography.

To examine changes in retinal thickness in the area affected by BRVO, OCT images with no ME (central macular thickness <300 μm) were analyzed by two authors (A.S, H.S). Based on FA findings at 12 months, the patients were divided into two groups: ischemic BRVO (NPAs >10 DAs); and nonischemic BRVO (NPAs ≤10 DAs). Based on the report of Lim et al. [18] we defined the BRVO-affected area in the superotemporal BRVO as the outer superior areas of the Early Treatment Diabetic Retinopathy Study (ETDRS) subfield in the Macular Cube 200 × 200 Combo protocol. We also defined the BRVO-affected area in the inferotemporal BRVO as the outer inferior areas of the ETDRS subfield in the Macular Cube 200 × 200 Combo protocol. Retinal thickness of the BRVO-affected area was defined as the mean distance between the ILM and RPE at the outer superior or inferior section as defined by the ETDRS grid, which was obtained using the Macular Cube 200 × 200 Combo protocol at each point of resolution of ME, and the retinal thickness of a symmetric area within the BRVO-affected area was defined as the retinal thickness of the control area (Fig 2). The retinal thicknesses of the ischemic or nonischemic BRVO-affected areas and control areas were used for analysis. The size of manually delineated ischemic areas in FA and thinning areas in OCT images within the macular area (6 mm × 6 mm) were measured using ImageJ software (National Institutes of Health, Bethesda, Maryland, USA; available at http://rsbweb.nih.gov) (Fig 3). Changes in thinning retina within the macular area (6 mm × 6 mm) were evaluated.

## Statistical analysis

All statistical analyses were performed using IBM SPSS Statistics ver. 21.0 (IBM Co., Armonk, NY, USA). Data correlations between the status of OCT parameters (ELM, EZ, IZ, and PROS length) and BCVA at 12 months or number of IVRs at 12 months were investigated using the Mann-Whitney U-test and Spearman rank-correlation test. The time-course changes in the retinal thinning area, CFT, and BCVA were analyzed in the Wilcoxon signed-rank test. The retinal thicknesses of the ischemic or nonischemic BRVO-affected areas and control areas were analyzed using the Mann-Whitney U-test. Furthermore, OCT parameters influencing VA and number of IVRs at 12 months were examined using multivariate analysis of variance. A p value of less than 0.05 was considered to represent a statistically significant difference.

## Results

### Clinical course

**Fig 4:**
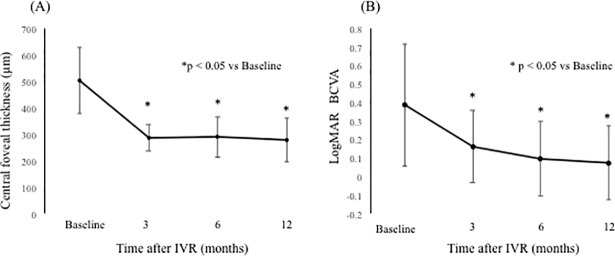
Changes in the mean central foveal thickness (CFT) and mean logarithm of minimum angle of resolution (logMAR) best-corrected visual acuity (BCVA) from baseline to 12 months after intravitreal ranibizumab injection (IVR). (A) CFT and (B) logMAR BCVA from baseline to 12 months after IVR. The CFT and visual acuity at 12 months were significantly improved compared with baseline (p < 0.05, respectively). logMAR = logarithm of minimum angle of resolution. IVR = intravitreal ranibizumab injection.

The baseline characteristics of patients are shown in Table 1. The mean logarithm of minimum angle of resolution best-corrected visual acuity (logMAR BCVA) at 3, 6, and 12 months was 0.16 ± 0.19, 0.09 ± 0.20, and 0.07 ± 0.20, respectively, which improved significantly from baseline at each visit (p < 0.0001, respectively) (Fig 4). The mean CFT at 3, 6, and 12 months was 286 ± 50 μm, 289 ± 76 μm, and 278 ± 83 μm, respectively, and significantly improved from baseline at each visit (p < 0.0001, respectively), while the mean number of IVRs at 6 and 12 months was 3.0 ± 1.3 and 3.9 ± 2.2, respectively. The mean number of IVRs for the first resolution of ME was 1.6 ± 0.8.

**Table 1:** Baseline patient characteristics.

No. of patients	27
Male, n (%)	13 (48)
Age (y), mean ± SD	62.8 ± 11.9
Duration of symptoms (weeks), mean ± SD	4.8 ± 3.2
Observation period (months), mean ± SD	21.8 ± 4.4
Baseline logMAR BCVA, mean ± SD	0.38 ± 0.33
Baseline CFT (μm), mean ± SD	502 ± 124
History of hypertension, n (%)	19 (70)

SD = standard deviation; logMAR = logarithm of minimum angle of resolution; BCVA = best-corrected visual acuity; CFT = central foveal thickness

At 12 months, 27 eyes underwent FA examination to determine the presence of NPAs. Fifteen eyes had NPAs, of which 12 had NPAs greater than >10 DAs and therefore scatter laser photocoagulation was performed. Three eyes with NPAs <10 DAs and 12 eyes without NPAs did not undergo scatter laser photocoagulation.

During the follow-up period, none of the patients showed adverse ocular events associated with IVR such as retinal detachment, endophthalmitis, and cataract. None showed neovascular glaucoma and vitreous hemorrhage concomitant with ischemia. One patient exhibited systemic adverse events, and 1 death occurred during the study.

### Relationship between outer retinal structure at the points of first resolution of ME and BCVA or number of IVRs at 12 months

**Fig 5:**
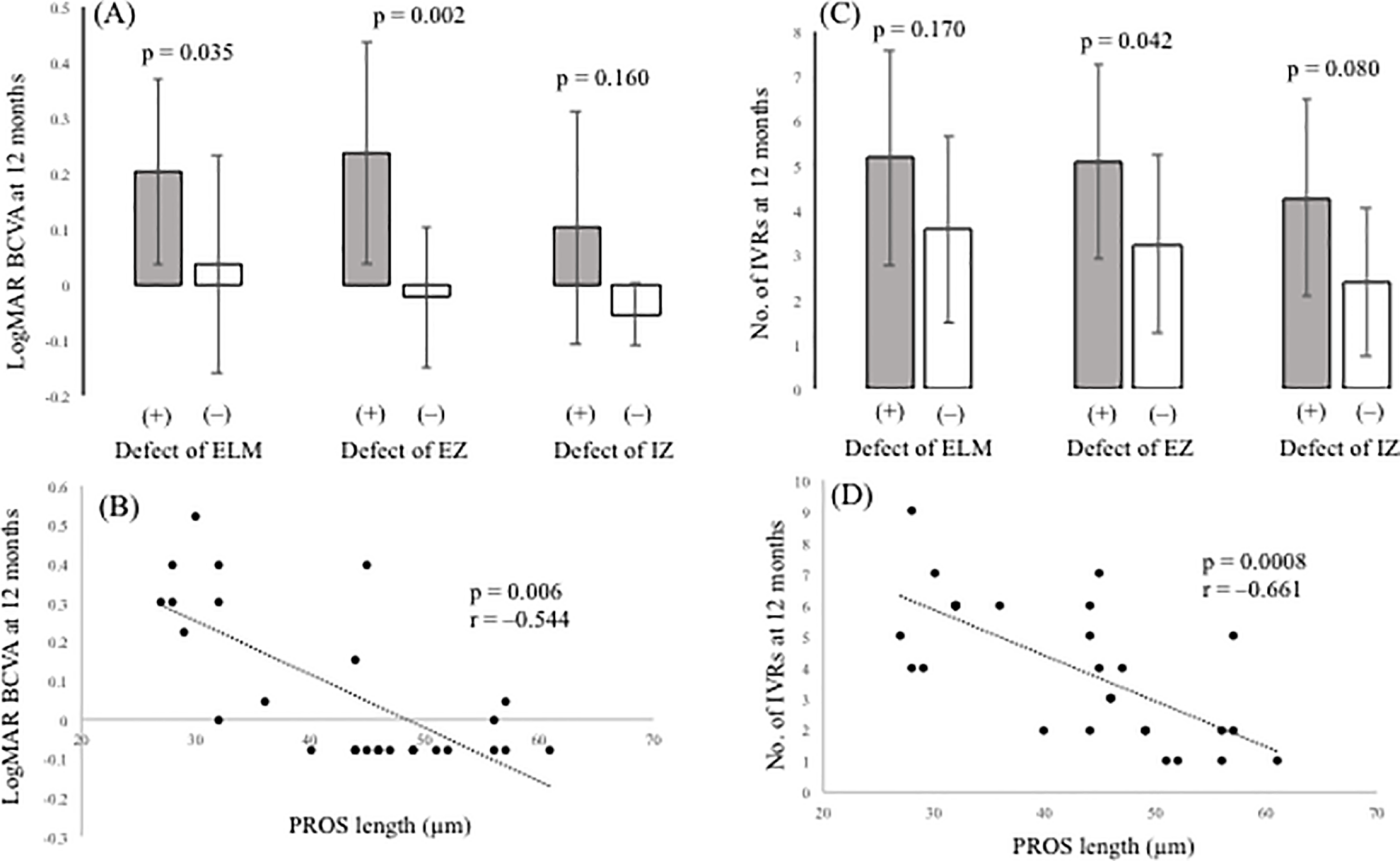
Relationship between optical coherence tomography (OCT) parameters and visual outcome or number of intravitreal ranibizumab injections (IVRs). (A) Correlation between logMAR BCVA at 12 months and the status of the ELM, EZ, and IZ. (B) Correlation between logMAR BCVA at 12 months and PROS length. (C) Correlation between the number of IVRs and the status of the ELM, EZ, and IZ. (D) Correlation between the number of IVRs and PROS length. OCT = optical coherence tomography; logMAR = logarithm of minimum angle of resolution; BCVA = best-corrected visual acuity; IVR = intravitreal ranibizumab injection; ELM = external limiting membrane; EZ = ellipsoid zone; IZ = interdigitation zone; PROS = photoreceptor outer segment.

At the points of first resolution of ME, defects in the ELM, EZ, and IZ were detected in 22.2% (6/27), 37.0% (10/27), and 81.4% (22/27) of patients, respectively. EZ defects did not completely resolve in any of the eyes, although PROS length could be measured in all. The mean PROS length at the points of first resolution of ME was 43.0 ± 10.3 μm. Fig 5 shows the relationship between outer retinal parameters at the points of first resolution of ME and BCVA or the number of IVRs at 12 months. Eyes with ELM and EZ defects at the points of first resolution of ME were correlated with a significantly lower BCVA at 12 months compared with eyes with preserved ELMs and EZs (p = 0.035, p = 0.002, respectively). However, eyes with IZ defects at the points of first resolution of ME were not correlated with a lower BCVA at 12 months compared with eyes with preserved IZs (p = 0.160). Defects in the EZ at the points of first resolution of ME significantly affected the number of IVRs at 12 months (p = 0.042), although defects in the ELM and IZ did not (p = 0.170, p = 0.080, respectively). PROS length at the points of first resolution of ME was significantly correlated with BCVA and number of IVRs at 12 months (p = 0.006, r = –0.544 and p = 0.0008, r = –0.661, respectively).

### Factors affecting BCVA and number of IVRs at 12 months in multivariate analysis

In univariate analysis, the EZ and PROS length at the points of first resolution of ME were significantly associated with BCVA and number of IVRs at 12 months. Multivariate analysis of variance was used to compare the EZ and PROS length. The results showed that PROS length at the points of first resolution of ME had the most significant effect on BCVA and number of IVRs at 12 months (p = 0.013, p = 0.012, respectively) (Table 2).

**Table 2:** Factors affecting BCVA and number of IVRs at 12 months in multivariate analysis.

Dependent variable	Independent variable	p value
BCVA	EZ	0.102
	PROS length	0.013
Number of IVRs	EZ	0.832
	PROS length	0.001

BCVA = best-corrected visual acuity; EZ = ellipsoid zone; PROS = photoreceptor outer segment; IVR = intravitreal ranibizumab

### OCT-based analysis of BRVO-affected areas

**Fig 6:**
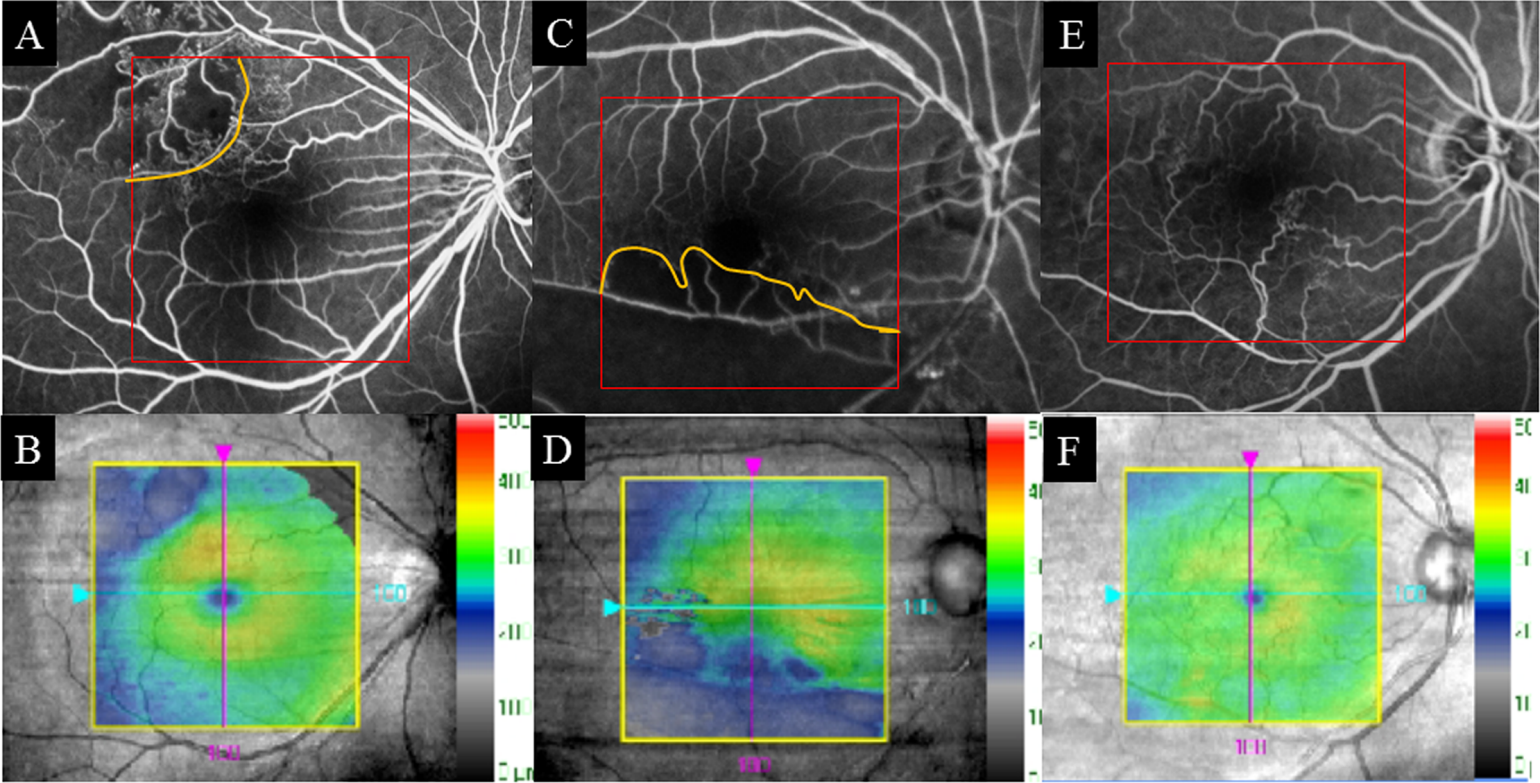
Fluorescein angiography (FA) images and color maps of retinal thickness using optical coherence tomography (OCT) images. (A, C) FA images of eyes with nonperfusion areas (NPAs) (shown in orange). (B, D) Color maps of eyes with NPAs which corresponded to the thinning areas (in blue) detected by OCT. (E) FA of eyes without NPAs. (F) The thinning areas were not detected in OCT.

**Fig 7:**
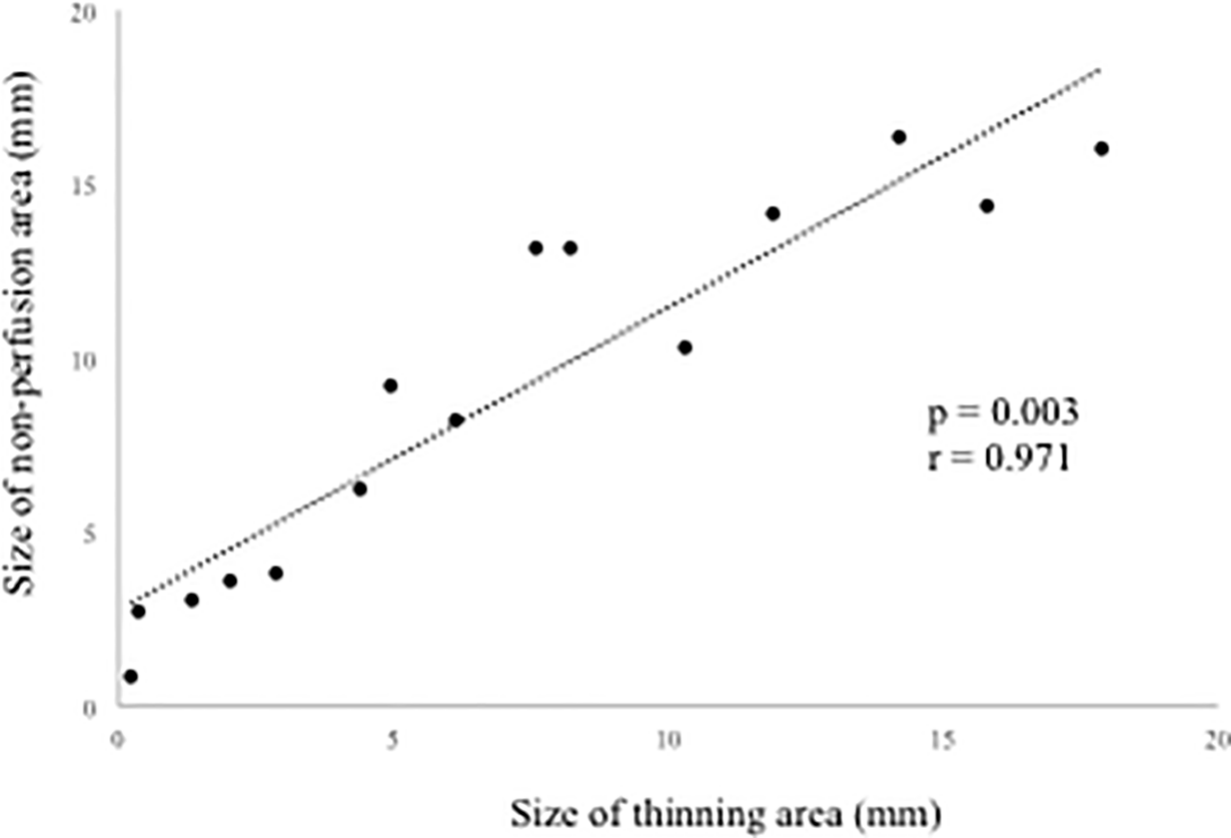
Correlation between nonperfusion areas (NPAs) in fluorescein angiography (FA) and thinning areas in optical coherence tomography (OCT).

**Fig 8:**
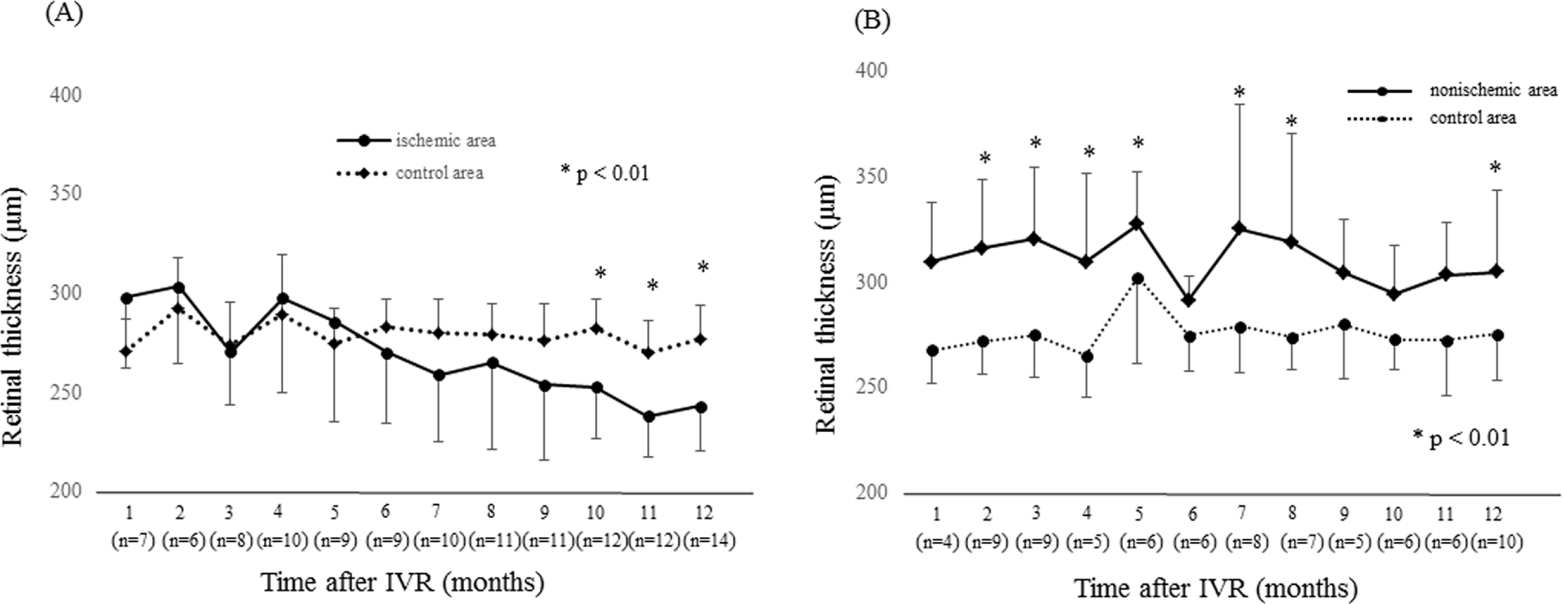
Changes in the retinal thickness of ischemic and nonischemic branch retinal vein occlusion (BRVO). (A) Changes in the retinal thickness of ischemic BRVO-affected areas and control areas at points of resolution of macular edema (ME) from 1 month to 12 months. Retinal thicknesses at point of recurrence of ME were excluded. Retinal thicknesses of ischemic areas after 10 months were significantly less than those of control areas (p < 0.01). (B) Changes in the retinal thickness of nonischemic BRVO-affected areas and control areas at points of resolution of ME from 1 month to 12 months. The retinal thickness of nonischemic areas tended to be greater than that of control areas. IVR = intravireal ranibizumab.

**Fig 9:**
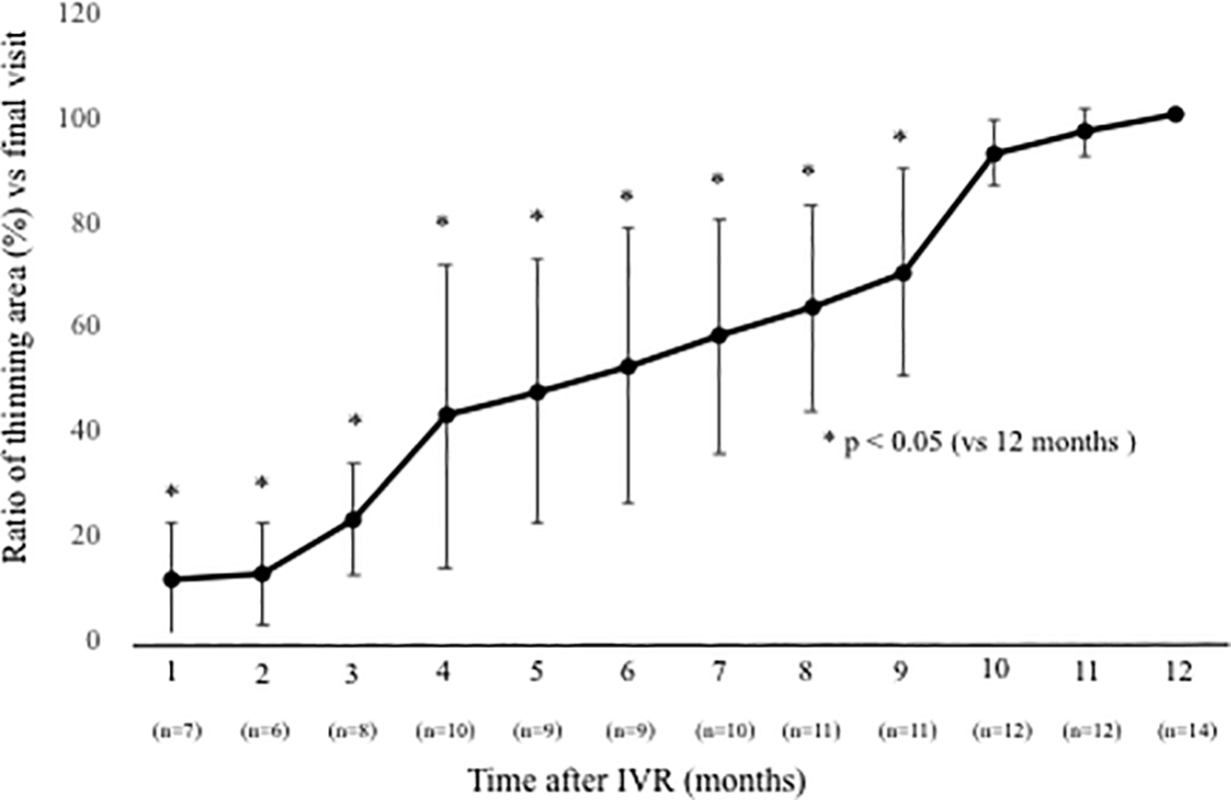
Changes in the ratio of thinning areas within the macular area at points of resolution of macular edema (ME) compared with the final visit. The ratio of the thinning area, which is indicated in blue on the macular cube scan (200 × 200), was defined as the measurement of the thinning area at each visit/measurement of the thinning area at the final visit. Only eyes that met the definition of resolution of ME are included. The ratio of thinning areas gradually increased until 9 months (p < 0.05, respectively). IVR = intravitreal ranibizumab.

NPAs were detected by FA in 15 eyes at 12 months. The NPAs in the macular area with no ME corresponded to the thinning areas detected by the OCT macular cube at 12 months. The thinning area was defined as that indicated in blue on the macular cube scan. On the other hand, in 12 eyes without NPAs, no thinning areas were detected using OCT (Fig 6). NPA size (9.02 ± 5.34 mm) on FA and thinning area (7.22 ± 5.72 mm) on OCT within the macular area (6 mm × 6 mm) showed a significant correlation (Fig 7, p = 0.003, r = 0.971). Changes in retinal thickness in BRVO-affected and control areas are shown in Fig 8. The retinal thickness of ischemic BRVO-affected areas after 10 months was significantly less than that of control areas (10 months, p = 0.001; 11 months, p = 0.005; 12 months, p = 0.003). The retinal thickness of nonischemic BRVO-affected areas at each time point was significantly greater than that of control areas (2 months, p = 0.007; 3 months, p = 0.007, 4 months, p = 0.043; 6 months, p = 0.046; 7 months, p = 0.035; 8 months, p = 0.042; 12 months, p = 0.028). The retinal thickness of ischemic BRVO-affected areas gradually thinned in most eyes, and a significant reduction was noted from 10 months and sustained until 12 months compared with baseline. Changes in the area of thinning retina within the macular area are shown in Fig 9. The thinning retina area gradually expanded in most eyes.

## Discussion

In this study, patients received IVRs on the 1+PRN regimen. VA and CFT significantly improved from baseline to 12 months. An improvement of 0.31 units on the logMAR scale with 3.9 injections during the 12-month period was seen. Ito et al. reported that a single intravitreal injection of bevacizumab (IVB; off-label use) achieved noninferior visual outcomes compared with those achieved by 3 monthly injections in eyes with ME secondary to BRVO[19]. Recently, Miwa et al. [9] have reported a comparison of 1 initial IVR (1+PRN) and 3 monthly IVRs (3+PRN) in patients with ME due to BRVO. In their report, the 1+PRN and 3+PRN regimens achieved similar clinical results, and the mean number of IVRs was 3.8 in the 1+PRN group and 4.6 in the 3+PRN group over 12 months. Our study also yielded comparable results (3.9 in this study), including VA and CFT. Narayanan et al. reported the comparative study of IVR vs IVB with a 6-month 3+PRN regimen (MARVEL study) [20]. In their report, there was no significant difference between the two groups in terms of VA, CFT, and the number of injections, and the mean IVR was 3.2 and mean IVB 3.0 over 6 months. More recently, Pichi et al. have conducted a comparative study of IVR vs aflibercept with the 1+PRN regimen[21]. They reported no significant difference between the two groups in terms of VA, CFT, and number of injections. They administered a mean of 2.8 IVRs and 2.6 intravitreal aflibercept injections over 12 months, comparable with the results of our study. The combined results suggest that the 1+PRN regimen may be a useful therapy for ME due to BRVO and require fewer injections. Minimizing the number of injections may contribute to reducing the risk of systemic complications associated with IVR. However, the current study had one treatment arm without a control group, and direct comparisons with the results of previous clinical trials of ranibizumab[3,4] cannot be made because of differences in the retreatment criteria and follow-up schedule.

For investigation of OCT-based prognostic factors, we examined elements of the outer retina such as the ELM, EZ, IZ, and PROS length, because several authors including us reported that the outer retina morphology is strongly correlated with visual function in patients with vitreoretinal disease[22–29]. Chang et al. [22] found that the postoperative VA was correlated with a restored ELM in patients with macular holes, while Ooka et al. [23] found that the preoperative lengths of ELM defects were significantly correlated with foveal sensitivity after macular hole surgery. Wakabayashi and associates[24] reported that restoration of the foveal ELM in the early postoperative period helped predict better visual outcome in patients with macular holes. Regarding the EZ and IZ, Cheng et al. [25] found that worsening VA was correlated with greater disruption of the EZ and IZ in patients with Behçet’s disease. According to Shimozono et al. [26], the status of the EZ and IZ is a useful prognostic factor of the results of epiretinal membrane surgery. Previously, it was reported that intact EZ and ELM and the presence of a foveal bulge at final follow-up are prognostic factors of resolution of ME and better visual improvement. [27,28] In terms of PROS length, Hasegawa et al. [29] noted that PROS length is a good parameter indicating the integrity of foveal photoreceptors in eyes with central serous chorioretinopathy. We also previously reported that PROS length is a strong indicator of visual function and prognostic factor in patients with epiretinal membrane[15]. However, it is occasionally difficult to analyze the outer retina precisely in cases of ME, because OCT signals deteriorate due to the presence of fluid and hemorrhage. Accordingly, we examined OCT images of the outer retina at points of first resolution of ME. In univariate analysis, EZ status and PROS length at first points of ME resolution were significantly correlated with BCVA and number of IVRs at 12 months. In the comparison of EZ and PROS length using multivariate analysis of variance, our results showed that PROS length has a higher correlation with BCVA and number of IVRs than the EZ. This suggests that PROS length is a better indicator of final visual outcome and number of IVRs. Previously, several authors noted that baseline BCVA had prognostic relevance for visual outcome in patients with BRVO treated with anti-VEGF therapy[16,30]. Recently, it has been reported that the number of IVBs in BRVO patients treated with the 1+PRN regimen was significantly less in the better VA group during 12 months. [31] We previously found that PROS length strongly correlated with BCVA, which reflected retinal function^15^. Those reports might support the suggested reason for the significant correlation between the status of the outer retina such as the EZ and PROS length and the number of IVRs.

With regard to analysis of retinal thickness in BRVO-affected areas, we found that ischemia-related thinning areas shown on OCT images reflected the NPAs obtained by FA. Several studies indicated that retinal ischemia associated with diabetic retinopathy and RVO induces thinning of the inner retina[32–34]. Ebneter et al. [35] reported that the thickness of the layers of the outer retina in the ischemic retinal area is well preserved, whereas that of the layers of the inner retina is reduced, in animal models of RVO. The inner retina receives its blood supply from the retinal blood vessels, whereas the outer retina receives its supply mainly from the choroidal circulation. In this study, although there was a trend for reduced retinal thickness between the ischemic BRVO-affected areas and control areas, a significant reduction in the ischemic BRVO-affected area was observed after 10 months. In patients with retinal artery occlusion, Chen et al. [36] confirmed diffuse thickening in the macular area in the acute stage. In contrast, retinal thinning was confirmed in the chronic central retinal artery occlusion phase. In BRVO, the timing of onset and thinning may differ from that in retinal artery occlusion. In nonischemic BRVO, retinal thinning was not confirmed at each time point. Thus, retinal thinning was caused by retinal ischemia, not by perfused BRVO alone.

To the best of our knowledge, this study is the first to demonstrate the clinical course of retinal thinning of NPAs. During observation, the retinal thickness of the ischemic BRVO-affected areas gradually thinned and expanded, and a few months were required for a significant change to be noted. This likely reflects the time required for the ischemia to damage the inner retina, and that damage subsequently became obvious in OCT analysis. More recently, several authors have reported that OCT angiography (OCTA) can detect NPAs[37–39]. Although OCTA is effective in detecting NPAs, OCT alone might be sufficient to detect NPAs. The present results suggest that OCT-based examination of BRVO-affected areas is a useful, safe examination of ischemia in patients with BRVO.

This study had some limitations. A relatively small number of eyes was examined, and the follow-up period was relatively short. Nevertheless, it was the first to demonstrate that the determination of PROS length is useful clinically, since it is an important structural feature that reflects visual function and predicts visual outcome and number of IVRs in patients with BRVO. Examination of thinning retinas during the clinical course can reflect the presence and progression of NPAs in patients with BRVO.

## Supporting information

S1 File Original protocol in Japanese. (PDF)

S2 File Original protocol in English. (PDF)

S3 File Representative case. (PDF)

## Abbreviations

OCT: optical coherence tomography
IVR: intravitreal ranibizumab
BRVO: branch retinal vein occlusion
BCVA: best-corrected visual acuity
SD-OCT: spectral domain optical coherence tomography
ELM: external limiting membrane
EZ: ellipsoid zone
IZ: interdigitation zone
PROS: photoreceptor outer segment
ME: macular edema
NPAs: nonperfusion areas
