# Supplementary material for: Optical coherence tomography findings as a predictor of clinical course in patients with branch retinal vein occlusion treated with ranibizumab
Source: PLoS One. 2018 Jun 20;13(6):e0199552. doi: 10.1371/journal.pone.0199552 (PMC6010278; doi:10.1371/journal.pone.0199552)
Supplement: S1 File — (PDF) [file pone.0199552.s001.pdf]

臨床試験実施計画書

課題名　　網膜静脈閉塞症のラニビズマブ治療患者におけるSD-OCTを用いた視力予後因子の検討

試験責任医師　　向後　二郎
所属　　聖マリアンナ医科大学眼科
住所　　川崎市宮前区菅生２－16－１
内線ＰＨＳ　　81281
FAX　　044-976-7523
電子メール　　kogo@marianna-u.ac.jp

試験実施期間　承認後　–　平成 28 年 12 月 31 日（3 年間）
作成日　　平成 25 年 10 月 21 日

1.　試験の背景

網膜静脈閉塞症（RVO）は、網膜静脈の閉塞部位の違いによって分類され、網膜静脈の分枝が閉塞した網膜静脈分枝閉塞症（BRVO）と視神経乳頭部の篩状板付近で網膜中心静脈が閉塞した網膜中心静脈閉塞症（CRVO）に大別される。網膜中心静脈が先天的に篩状板部で上下 2 本に分枝している場合があり、うち 1 本が閉塞した場合を半側網膜静脈閉塞症（HRVO）という。いずれの場合も黄斑部に浮腫が至ると、視力は著しく低下をきたす。

RVO の発症するメカニズムとして、網膜静脈が閉塞されて静脈灌流が阻害されることにより網膜が低酸素状態になるとともに、閉塞部位から末梢側の灌流域の毛細血管圧が上昇し血管網膜関門が破綻するといわれており、これらによって、血管透過性が亢進し黄斑浮腫が生じると考えられている。血管透過性を強く亢進させる要因として、網膜が低酸素状態となることによって誘発される VEGF が強く関与していることが示唆されている。

一方、日本における代表的な疫学研究である久山町研究の報告より、RVO の発症リスクファクターとして、高血圧、慢性腎臓疾患があり、また男性では 40 代以降、女性では 50 代以降に、加齢とともに発症率が増加する。日本人の発症率は 40 歳以上で 2.1%、有病率は他のアジア人や白人より高いと報告されている。

RVO に対する治療として主に行われてきたのが抗 VEGF 薬治療、ステロイド治療、レーザー光凝固、硝子体手術である。ただ抗 VEGF 薬治療については適応外使用のみで承認された薬剤はなかった。そこで本年本邦で初めて抗 VEGF 薬としてラニビズマブは RVO に対して承認された。ラニビズマブは RVO に伴う黄斑浮腫に対する国内及び海外での第Ⅲ層試験で、有効性と安全性が検証されている。しかしながら、本邦における実臨床下での研究報告はない。

光干渉断層計（SD-OCT）の普及により視細胞外節/内節（IS/OS）や外境界膜のような解剖学的所見が得られるようになった。いくつかの OCT による研究では眼疾患における質的因子の評価が行われている。糖尿病黄斑浮腫（DME）の患者では最高矯正視力（BCVA）と視細胞外節長（PROS length）と相関している。また萎縮型加齢黄斑変性症（Dry AMD）の患者において外層の厚み、範囲、体積と BCVA が相関する。黄斑円孔の患者では外中心窩厚と外顆粒層（ONL）の相対的な反射が BCVA と相関している。AMD では ONL の体積が BCVA と相関している。我々は特発性黄斑上膜（ERM）の患者では PROS length が BCVA と相関していることを報告した。

本邦における RVO に対するラニビズマブの視力維持改善効果の検討及び SD-OCT を用いた視力予後規定因子の詳細な検討は臨床的意義が高い可能性がある。

2.　試験の目的と必要性

本試験では RVO に対し、ラニビズマブを投与し、その治療効果と視力予後規定因子について検討する。

3.　試験薬の概要

ラニビズマブは、VEGF に対するヒト化モノクローナル抗体の Fab 断片であり、硝子体内への投与を目的に硝子体内投与専用薬剤として開発された。

作用機序として、VEGF 分子中の VEGF 受容体結合ドメインに特異的かつ高親和性に結合することで、VEGF の血管透過性の亢進作用及び血管新生作用を抑制する。

投与方法としては、ラニビズマブ 0.5mg（0.05ml）を硝子体内投与する。

4.　対象患者

（1）選択基準

①　当院眼科外来を受診し、RVO と診断された患者

②　18 歳以上で本試験の参加に同意が得られた患者

③　少数視力が 0.1 以上の患者

④　平均中心窩網膜厚が 250μm 以上の患者

（2）除外基準

①　本試験開始前 4 カ月以内にレーザー光凝固術を施行した患者

②　本試験開始前 3 ヵ月以内にステロイド硝子体内投与を行った患者

③　本試験開始前 3 ヵ月以内に抗 VEGF 薬の硝子体内投与もしくは 6 カ月以内に抗 VEGF 薬の全身投与を行った患者

④　本試験開始前 3 ヵ月以内に脳卒中や心筋梗塞の既往がある患者

⑤　妊娠中もしくは妊娠や授乳の可能性がある患者

⑥　重度の肝障害や重度の CKD 患者/透析が必要な患者

⑦　本試験開始前 6 カ月以内でコントロール不良（HbA1c＞10）であった糖尿病の患者

⑧　本試験開始前 6 カ月以内でコントロール不良（血圧≧160/100 ㎜ Hg）であった高血圧の患者

⑨　網膜硝子体病変や角膜病変、ぶどう膜炎、緑内障等の RVO 以外の眼疾患で対象眼に視力障害を有する患者

5.　患者の同意

研究担当医師は対象となる患者に下記の事項を別添の説明文書で十分に説明し、患者よりこの試験の参加について文書で同意を得る。

1. 臨床試験についての説明
2. この試験の目的
3. 薬剤ならびに治療の方法
4. この試験の予定参加期間
5. この試験への予定参加人数
6. この試験薬の予想される効果と起こるかもしれない副作用
7. この薬を使用しない場合の、他の治療方法
8. この試験中に、あなたの健康に被害が生じた場合について
9. この試験への参加について
10. プライバシーの保護
11. あなたの費用負担について
12. 治療に関する問い合わせ・質問などについて

6.　試験の方法

(1)　試験のデザイン　介入研究

(2)　試験のアウトライン　ラニビズマブを投与し、その治療効果と SD-OCT を用いて視力予後規定因子を検討する

(3)　被験者の試験参加期間　投与後 1 年間

(4)　試験薬の用法　ラニビズマブ 2.3mg/0.23ml を 0.05ml（0.5mg）硝子体腔へ投与する。毎月 1 度の観察の基必要に応じて投与を行う。

(5)　追加投与基準　OCT にて網膜下液や網膜浮腫などの滲出性変化を認めた際に追加投与を行う。

7.　予定症例数

50 例を予定している。

8.　評価項目

(1)　主要評価項目

１２ケ月後矯正視力　（logMAR 視力で０．３以上の改善で治療効果ありと判断）

(2)　副次的評価項目

・網膜厚　（OCT にて治療前より２００μm 以上の減少で治療効果ありと判断）

・FA での蛍光漏出の変化および無潅流領域の変化

・眼圧

・眼底所見（血管の蛇行、出血の程度）
・投与回数
・視力予後規定因子（上記副次的評価項目と１２ケ月後矯正視力で多変量解析を行う）

9.　観察項目

治療開始前	視力、蛍光眼底造影検査、眼底写真<br>眼圧、OCT、視野検査
薬剤投与　翌日	眼圧、眼底検査
毎月	視力、眼圧、眼底、OCT、蛍光眼底造影検査（6 カ月毎）、視野検査（6 カ月毎）

※OCT:　網膜光干渉断層計　非侵襲的な網膜黄斑部の断層解析装置である。

10. 中止基準

参加者が臨床研究からの離脱を希望した場合。予期せぬ重篤な合併症、感染症、眼内炎などの重篤な合併症が生じた場合にはそれぞれの病態に応じた治療を直ちに開始する。必要に応じて本試験は中止とする場合もあるが分析の対象には含まれる。

11. 有害事象発生時の取り扱い

（１）　有害事象の定義

①　原因不明の視野欠損。
②　注射直後の急激な視力低下（LogMAR 視力で 0.3 以上の低下)。
③　注射直後の急激な眼圧上昇
④　眼内炎

（２）　有害事象の評価と報告

①　緑内障点眼・内服・点滴治療にも関わらず、眼圧上昇が 20mmHg 以上続くもの
②　LogMAR 視力で 0.3 以上の著明な視力低下
③　動的量的視野検査にて、術前より視野が 30%以上欠損しているもの
④　前房蓄膿を伴う眼内炎の発症

上記の何れかが見られた場合は直ちに適切な処置を行うとともに、カルテに齟齬なく記載する。
試験責任医師は、重篤な有害事象の発生を認めたときは、速やかに生命倫理委員長及び、臨床試験部会に報告する。

（３）予想される有害事象

高眼圧症、眼内炎、原因不明の視力低下、視野欠損
ただし、現時点においてこのような有害事象の報告はなく、極めて少ないと考えられる

12. 試験実施期間

承認後-平成 27 年 12 月 31 日

13. 被験者の人権および安全性・不利益に対する配慮

患者の個人情報は個人情報管理者が匿名化を行い管理する。本試験の結果は学会や医学雑誌などに公表されることがあるが、情報は匿名化されるために患者の氏名が明らかになることはない。

14. 患者の費用負担

診療に掛かる費用、(薬費、処置料、点眼薬等薬剤費、入院費、定期的な経過観察目的の眼底検査や蛍光眼底造影検査などの費用）は、通常の保険請求を行う。

15. 健康被害の補償および保険への加入

ラニビズマブを含めた医薬品を適正使用したにも関わらず、医薬品による副作用や明らかな過失によるものであると確認された場合は、個別に話し合いを持ち補償を行う可能性がある。副作用によって一定以上の健康被害が生じた場合、医薬品副作用被害救済制度による救済を受けることができる。担当医師個人の医療過失保険への加入はある。なお、本試験については、患者に生じた、本試験と因果関係を否定できない健康被害の補償に備えて、研究責任医師等本研究に携わるすべての者を被保険者として臨床研究保険に加入する。

この保険は、本試験に起因して、研究期間中または終了後 1 年以内に患者に健康被害（死亡または医薬品副作用被害救済制度基準の後遺障害 1 級または 2 級の重篤な身体障害）が生じた場合に、研究責任医師等が負担する補償責任、または本試験に起因して患者に身体障害が生じた場合に法律上の賠償責任を負担することによって被る損害に対し保険金を支払うものである。ただし、本研究において、委託者であるノバルティス ファーマ株式会社はいかなる補償責任または賠償責任も負わないものとする。

16. 「世界医師会ヘルシンキ宣言」、および「臨床研究に関する倫理指針」を遵守して本研究を行う。

17. 記録の保存

登録通し番号と患者 ID は対応表を作り、別個に保管する。保管するデータは、視力(LogMAR)、中心窩網膜厚、眼圧、蛍光眼底造影検査所見、及び年齢、性別、である。実施責任者がこれを保管する。

保管期間：試験開始後から試験終了後 2 年間

保管場所：眼科医局内

18. 研究結果の公表

研究結果は、プライバシーの保護に十分配慮した上で、国内外の学会・雑誌で発表予定である。

19. 研究組織

聖マリアンナ医科大学病院

聖マリアンナ医科大学　眼科学教室

神奈川県川崎市宮前区菅生２－16－１（代表電話　044-977-8111, 内線 3252）

研究責任者：眼科学		向後二郎	講師	（内線 3252　PHS 81281）
研究分担者：	同	高木　均	教授	（内線 3252　PHS 80810）
	同	徳田直人	講師	（内線 3252　PHS 81535）
	同	塩野　陽	大学院生	（内線 3252　PHS 81722）
	同	三井一央	大学院生	（内線 3252　PHS 80797）
	同	佐々木寛季	大学院生	（内線 3252　PHS 81699）
個人情報管理者：眼科学		宗正泰成	講師	（内線 3252　PHS 81156）

20. 参考資料・文献

1. Arakawa S, et al, IOVS 2011:52:5905-9. Nine-year incidence and risk factors for retinal vein occlusion in a general Japanese population: the Hisayama Study.
2. Yasuda M., et al, IOVS 2010:51 :3205-9 Prevalence and systemic risk factors for retinal vein occlusion in a general Japanese population: the Hisayama study.
3. Forooghian F, et al. Retina 2010 ;30 :63-70. Relationship between photoreceptor outer segment length and visual acuity in diabetic macular edema.
4. Pappuru RR, et al. IOVS 2011;52 :6743-8. Relationship between outer retinal thickness substructures and visual acuity in eyes with dry age-related macular degeneration.
5. Kashani AH, et al. IOVS 2009;50:3366-73. Quantitative subanalysis of cystoid spaces and outer nuclear layer using optical coherence tomography in age-related macular degeneration.
6. Christensen UC, et al. Br J Ophthalmol 2010;94:41-7. Macular morphology and visual acuity after macular hole surgery with or without internal limiting membrane peeling.
7. Shimozono M, et al. Graefes Arch Clin Exp Ophthalmol 2011 ;249 :1469-76. Restoration of the photoreceptor outer segment and visual outcomes after macular hole closure: spectral-domain optical coherence tomography analysis.
8. Shiono A, et al. Ophthalmology. 2013 Apr;120(4):788-94. Photoreceptor outer segment length: a prognostic factor for idiopathic epiretinal membrane surgery.
