# Supplementary material for: Optical coherence tomography findings as a predictor of clinical course in patients with branch retinal vein occlusion treated with ranibizumab
Source: PLoS One. 2018 Jun 20;13(6):e0199552. doi: 10.1371/journal.pone.0199552 (PMC6010278; doi:10.1371/journal.pone.0199552)
Supplement: S2 File — (PDF) [file pone.0199552.s002.pdf]

Project summary

To examine the relationship between optical coherence tomography (OCT) images and clinical course in eyes with branch retinal vein occlusion (BRVO) treated with intravitreal ranibizumab (IVR). Correlations between best-corrected visual acuity (BCVA) or number of IVRs after 12 months and OCT parameters including photoreceptor outer segment (PROS) length at first resolution of macular edema (ME) were assessed. OCT parameters influencing BCVA and number of IVRs were evaluated using multivariate analysis. Correlations between nonperfusion areas (NPAs) and thinning areas and changes in retinal thickness of BRVO-affected areas were assessed.

Protocol title:

To Investigate Prognostic Factors for Visual Improvement in Patients Undergoing Intravitreal Ranibizumab for Retinal Vein Occlusion (**ClinicalTrials.gov Identifier:** NCT02144662)

Research contact person and Public contact person:

Principal investigator: Jiro Kogo, M.D., Ph.D.

Department of Ophthalmology, St. Marianna University School of Medicine, 2-16-1 Sugao, Miyamae-ku, Kawasaki, Kanagawa, Japan

Tel: +81-44-977-8111

Fax: +81-44-976-7435

E-mail: kogo@marianna-u.ac.jp

Rationale & background information

BRVO is a retinal vascular disease and cause of visual loss due to macular edema (ME) and retinal ischemia. The Branch Vein Occlusion Study (BVOS) reported that grid laser photocoagulation increased visual acuity in patients with ME due to BRVO[1]. However, that effect was limited. In 2009, the Standard Care vs Corticosteroid for Retinal Vein Occlusion (SCORE) study showed that intravitreal triamcinolone administration resulted in similar visual improvement but with high rates of intraocular pressure elevation.[2] Detailed information on macular morphology,

such as the photoreceptor inner segment/outer segment (IS/OS) junction and external limiting membrane (ELM), can be obtained in spectral domain-optical coherent tomography (SD-OCT). Some OCT studies evaluated other quantitative factors in eye disease. It was reported that PROS length was correlated with best-corrected visual acuity (BCVA) in patients with diabetic macular edema.[3] Other investigators suggested that the thickness, area, and volume of the outer layer were correlated with BCVA in patients with dry age-related macular degeneration (AMD).[4] Outer foveal thickness (OFT) and relative reflectivity of the outer nuclear layer (ONL) were associated with BCVA in patients with macular hole.[5,6] The volume of the ONL was found to be associated with BCVA in patients with AMD.[7] The aim of this study was to investigate the pretreatment quantitative factors as shown in SD-OCT images that correlate with posttreatment VA in patients who underwent intravitreal Lucentis (Ranibizumab) for BRVO.

References

1. The Branch Vein Occlusion Study Group. Argon laser photocoagulation for macular edema in branch vein occlusion. Am J Ophthalmol. **98**, 271–282 (1984).
2. Scott, I. U. et al. A randomized trial comparing the efficacy and safety of intravitreal triamcinolone with standard care to treat vision loss associated with macular edema secondary to branch retinal vein occlusion: the Standard Care vs Corticosteroid for Retinal Vein Occlusion (SCORE) study report 6. Arch Ophthalmol. **127**, 1115–1128 (2009).
3. Forooghian F, et al. Retina 2010 ;30 :63-70. Relationship between photoreceptor outer segment length and visual acuity in diabetic macular edema.
4. Pappuru RR, et al. IOVS 2011;52 :6743-8. Relationship between outer retinal thickness substructures and visual acuity in eyes with dry age-related macular degeneration.
5. Christensen UC, et al. Br J Ophthalmol 2010;94:41-7. Macular morphology and visual acuity after macular hole surgery with or without internal limiting membrane peeling.

6. Shimozono M, et al. Graefes Arch Clin Exp Ophthalmol 2011 ;249 :1469-76. Restoration of the photoreceptor outer segment and visual outcomes after macular hole closure: spectral-domain optical coherence tomography analysis.
7. Kashani AH, et al. IOVS 2009;50:3366-73. Quantitative subanalysis of cystoid spaces and outer nuclear layer using optical coherence tomography in age-related macular degeneration.

Study goals and objectives

Primary Objective: To investigate the pretreatment quantitative factors as shown in SD-OCT images those correlate with posttreatment VA in patients who underwent intravitreal Lucentis (Ranibizumab) for BRVO.

Secondary Objectives: Correlations between posttreatment BCVA and pretreatment factors were evaluated, including age, pretreatment BCVA, photoreceptor outer segment (PROS) length, central foveal thickness (CFT), outer foveal thickness (OFT), and outer nuclear layer thickness (ONLT). The factors influencing posttreatment BCVA were evaluated using multiple regression analysis.

Study Design

Intervention Model: Single Group Assignment
Masking: None (Open Label)
Primary Purpose: Treatment

Methodology

<u>Treatment</u>

All patients received 1 initial intravitreal ranibizumab followed by a monthly PRN without a loading phase. At monthly visits, repeat injections were administered if there was evidence of ME (mean central foveal thickness >300 µm) or subretinal fluid (SRF) at the fovea on OCT images. Eyes with nonperfused areas >10 disc areas in size on fluorescein angiography (FA) were determined to represent ischemic BRVO.

Examination

Patients were scheduled for monthly monitoring visits during which they underwent a complete examination including measurement of logMAR BCVA with a Landolt chart, determination of intraocular pressure, and OCT (Cirrus HD-OCT, Carl Zeiss Meditec, Dublin, CA). Macular morphologic evaluation was determined based on OCT images at each visit. CFT was defined as the mean distance between the ILM and retinal pigment epithelium (RPE) within a central subfield. CFT measurements were derived from the software provided by the manufacturer. Examination of the outer retina including ELM, ellipsoid zone (EZ), interdigitation zone (IZ), and PROS length were examined with the Cirrus HD-OCT using 5-line raster scans. To assess retinal perfusion status, all patients underwent FA at 12 months.

The study aimed to identify prognostic factors for the improvement of BCVA and number of IVRs at 12 months, such as OCT parameters of the outer retina at the points of the first resolution of ME. These were examined using multivariate analysis. Resolution of ME was defined as CFT <300 μm and the absence of SRF.

To examine changes in retinal thickness in the area affected by BRVO, OCT images with no ME (central macular thickness <300 μm) were analyzed. Based on FA findings at 12 months, the patients were divided into two groups: ischemic BRVO (NPAs >10 disc diameters); and nonischemic BRVO (NPAs ≤10 disc diameters). We defined the BRVO-affected area in the superotemporal BRVO as the outer superior areas of the ETDRS subfield in the Macular Cube 200 × 200 Combo protocol. We also defined the BRVO-affected area in the inferotemporal BRVO as the outer inferior areas of the ETDRS subfield in the Macular Cube 200 × 200 Combo protocol. Retinal thickness of the BRVO-affected area was defined as the mean distance between the ILM and RPE at the outer superior or inferior section as defined by the ETDRS grid, which was obtained using the Macular Cube 200 × 200 Combo protocol at each point of resolution of ME, and the retinal thickness of a symmetric area within the BRVO-affected area was defined as the retinal thickness of the control area. The retinal thicknesses of the ischemic or nonischemic BRVO-affected areas and control areas were used for analysis. The size of manually delineated ischemic areas in FA and thinning areas in OCT images within the macular area (6 mm × 6 mm) were measured using ImageJ software (National

Institutes of Health, Bethesda, Maryland, USA; available at http://rsbweb.nih.gov). Changes in the area of thinning retina within the macular area (6 mm × 6 mm) were evaluated.

Eligibility

Ages Eligible for Study:	18 Years and older	(Adult, Senior)
Sexes Eligible for Study:	All	
Accepts Healthy Volunteers:	No	

Criteria

Inclusion Criteria:

- Age ≥ 18, Patient of RVO, BCVA ≥ 0.1 (decimal visual acuity), Mean VRT≥ 250 µm
- signed informed consent

Exclusion Criteria:

- Laser photocoagulation for ME ≤ 4 months prior to initiation of this study therapy
- Intraocular corticosteroid use ≤ 3 months prior to initiation of this study therapy
- History of anti-VEGF treatment (intravitreal ≤3 months prior to initiation of this study therapy, systemic ≤ 6 months prior to initiation of this study therapy)
- Stroke or myocardial infarction ≤ 3 months prior to initiation of this study
- Pregnancy or potential pregnancy, and breastfeeding
- Severe liver dysfunction, severe CKD/hemodialysis, uncontrolled DM (HbA1c>10), uncontrolled hypertension (BP≥ 160/100 mmHg)
- central retinal vein occlusion; hemi-central retinal vein occlusion; and other chorioretinal disease such as diabetic retinopathy, hypertensive retinopathy, and choroidal neovascularization.

Safety Considerations

Possible complications and side effects of the procedure include glaucoma (increased pressure in the eye) and endophthalmitis. Any of these rare complications may lead to severe, permanent loss of vision. Additional procedures may be needed to treat these complications. We may require medical or surgical intervention to prevent these complications. During the follow-up, we check for possible side effects and the results will discuss with patients. The Investigator is responsible for ensuring that all AEs that are observed during the study are recorded on the patient's clinical record.

Follow-Up

Date of protocol fixation: Mar /22/2014
Trial start date: 11/05/2013
Last follow-up date: August/24//2017

Statistical Analysis

All statistical analyses were performed using IBM SPSS Statistics ver. 21.0 (IBM Co., Armonk, NY, USA). A p value of less than 0.05 was considered to represent a statistically significant difference. Data correlations between final BCVA and SD-OCT characteristics or clinical features were investigated using the Spearman rank-correlation test.

Ethics

This study was approved by the Institutional Review Committee of St. Marianna University School of Medicine, and written informed consent for participation was obtained from all patients. The procedure used conformed to the tenets of the Declaration of Helsinki.

Support for the Project

This work received funding support from Novartis Pharma K.K.
