# Supplementary material for: Optical coherence tomography findings as a predictor of clinical course in patients with branch retinal vein occlusion treated with ranibizumab
Source: PLoS One. 2018 Jun 20;13(6):e0199552. doi: 10.1371/journal.pone.0199552 (PMC6010278; doi:10.1371/journal.pone.0199552)
Supplement: S3 File — (PDF) [file pone.0199552.s003.pdf]

Representative images

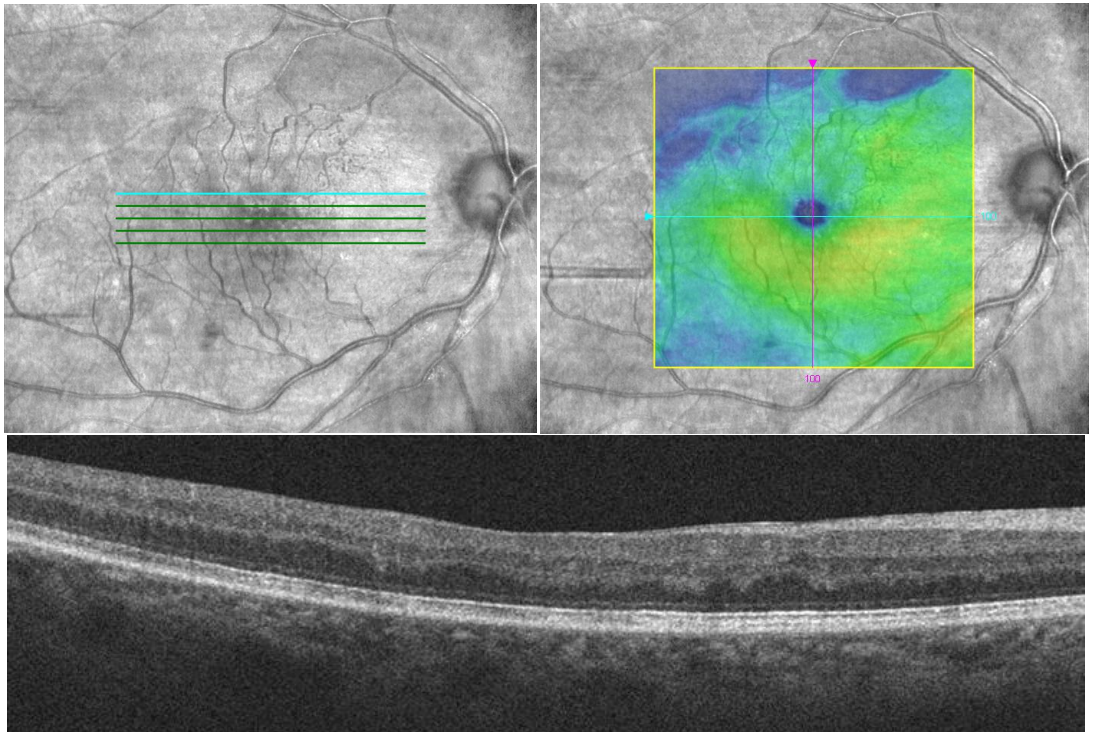

Representative images
As you can see, the most upper line of 5L did not cover the thinning area.
